# Increased Risk of Vascular Events in Emergency Room Patients Discharged Home with Diagnosis of Dizziness or Vertigo: A 3-Year Follow-Up Study

**DOI:** 10.1371/journal.pone.0035923

**Published:** 2012-04-27

**Authors:** Ching-Chih Lee, Hsu-Chueh Ho, Yu-Chieh Su, Brian C-H Chiu, Yung-Cheng Su, Yi-Da Lee, Pesus Chou, Sou-Hsin Chien, Yung-Sung Huang

**Affiliations:** 1 Community Medicine Research Center and Institute of Public Health, National Yang-Ming University, Taipei, Taiwan; 2 Department of Otolaryngology, Buddhist Dalin Tzu Chi General Hospital, Chiayi, Taiwan; 3 Division of Cardiology, Department of Internal Medicine, Buddhist Dalin Tzu Chi General Hospital, Chiayi, Taiwan; 4 Division of Hematology-Oncology, Department of Internal Medicine, Buddhist Dalin Tzu Chi General Hospital, Chiayi, Taiwan; 5 Cancer Center, Buddhist Dalin Tzu Chi General Hospital, Chiayi, Taiwan; 6 Department of Emergency Medicine, Buddhist Dalin Tzu Chi General Hospital, Chiayi, Taiwan; 7 Division of Neurology, Department of Internal Medicine, Buddhist Dalin Tzu Chi General Hospital, Chiayi, Taiwan; 8 Center for Clinical Epidemiology and Biostatistics, Buddhist Dalin Tzu Chi General Hospital, Chiayi, Taiwan; 9 Division of Plastic Surgery, Department of Surgery, Buddhist Dalin Tzu Chi General Hospital, Chiayi, Taiwan; 10 School of Medicine, Tzu Chi University, Hualian, Taiwan; 11 Department of Health Studies, University of Chicago, Chicago, Illinois, United States of America; Virginia Commonwealth University, United States of America

## Abstract

**Background:**

Dizziness and vertigo symptoms are commonly seen in emergency room (ER). However, these patients are often discharged without a definite diagnosis. Conflicting data regarding the vascular event risk among the dizziness or vertigo patients have been reported. This study aims to determine the risk of developing stroke or cardiovascular events in ER patients discharged home with a diagnosis of dizziness or vertigo.

**Methodology:**

A total of 25,757 subjects with at least one ER visit in 2004 were identified. Of those, 1,118 patients were discharged home with a diagnosis of vertigo or dizziness. A Cox proportional hazard model was performed to compare the three-year vascular event-free survival rates between the dizziness/vertigo patients and those without dizziness/vertigo after adjusting for confounding and risk factors.

**Results:**

We identified 52 (4.7%) vascular events in patients with dizziness/vertigo and 454 (1.8%) vascular events in patients without dizziness/vertigo. ER patients discharged home with a diagnosis of vertigo or dizziness had 2-fold (95% confidence interval [CI], 1.35–2.96; *p*<0.001) higher risk of stroke or cardiovascular events after adjusting for patient characteristics, co-morbidities, urbanization level of residence, individual socio-economic status, and initially taking medications after the onset of dizziness or vertigo during the first year.

**Conclusions:**

ER patients discharged home with a diagnosis of dizziness or vertigo were at a increased risk of developing subsequent vascular events than those without dizziness/vertigo after the onset of dizziness or vertigo. Further studies are warranted for developing better diagnostic and follow-up strategies in increased risk patients.

## Introduction

Dizziness or vertigo is a common reason for visiting the emergency room (ER), accounting for approximately 10% of total ER visits in the Unites States and more than $1.6 billion in health expenditures each year [1], [2], [3]. Although most patients with vertigo or dizziness are discharged, a potentially serious underlying disease, such as cerebrovascular events or myocardial infarction, may be overlooked [4], [5], [6].

Findings on the risk of stroke associated with vertigo or dizziness have not been consistent. A follow-up study of 121 patients found that up to 5% of them had major morbidity such as stroke within six months [7]. Kim et al. [8] reported that only 0.93% of ER patients discharged home with a diagnosis of vertigo or dizziness experienced a major vascular event during a follow-up of 180 days. In a population-based study, Kerber et al. [9] also reported a low proportion of cerebrovascular events in patients presenting with dizziness, vertigo or imbalance. However, several case reports and a study by Chase et al. suggested that stroke or vascular events are likely to happen among the vertigo or dizziness patients [5], [10], [11], [12].

The purpose of this study is to examine the incidence of vascular events in such patients identified through the National Health Insurance Research Database (NHIRD) in Taiwan. This allows for a comparison of the risk of vascular events between in ER patients discharged home with a diagnosis of dizziness or vertigo and those without [13].

## Materials and Methods

### Ethics statement

This study was initiated after approval by the Institutional Review Board of Buddhist Dalin Tzu Chi General Hospital, Taiwan. Since all identifying personal information was stripped from the secondary files before analysis, the review board waived the requirement for written informed consent from the patients involved.

### Database

The National Health Insurance program, which provides compulsory universal health insurance, was implemented in Taiwan in 1995. It enrolls up to 99% of the Taiwanese population and contracts with 97% of all medical providers [14]. The database contains comprehensive information on insured subjects, including dates of clinical visits, diagnostic codes, details of prescriptions and expenditure amounts. This study used the Longitudinal Health Insurance Dataset for 2004–2006 released by the Taiwan Nation Health Research Institute. The patients studied did not differ statistically significantly from the larger cohort in age, gender or health care costs, as reported by the Taiwan National Health Research Institute (http://w3.nhri.org.tw/nhird//date_cohort.htm) [15], [16].

### Study Population

**Figure 1:**
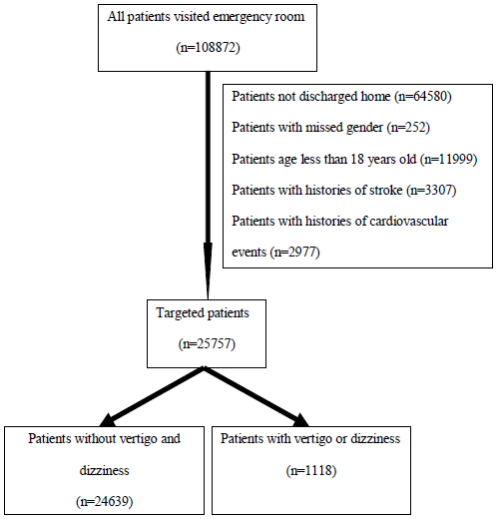
Flow diagram of the population-based study.

We identified patients older than 18 years who visited the ER in 2004. We excluded those with any type of stroke (International Classification of Diseases, 9th revision - Clinical Modification [ICD-9-CM] codes 430–438), acute myocardial infarction (ICD-9-CM 410), unstable angina (ICD-9-CM 411) or ventricular arrhythmia (ICD-9-CM 427.1, 427.4, 427.5 or 427.69) diagnosed before or during the index ER visit. After exclusion, we identified 1118 ER patients discharged home with a diagnosis of dizziness or vertigo (ICD-9-CM 780.4 and 386.0–386.9) and 24639 ER patients without a dizziness or vertigo diagnosis. Each patient was tracked for three years from his or her first discharge in 2004 to identify outcomes including hemorrhagic or ischemic stroke diagnosis codes (ICD-9-CM 430, 431, 433.×1, 434.×1, or 436.×), acute myocardial infarction (ICD-9-CM 410), unstable angina (ICD-9-CM 411) or ventricular arrhythmia (ICD-9-CM 427.1, 427.4, 427.5 or 427.69). To maximize case ascertainment, only patients hospitalized for the above vascular events were included. These patients were then linked to the administrative data for the period 2004–2006 to calculate cardiovascular or cerebrovascular disease-free survival time, with cases censored for patients who drew back guarantees from the National Health Insurance Program or were still robust without defined events at end of follow-up (Figure 1).

Medications which could result in dizziness or vertigo before ER visit were included for analysis. Medications consisted of anticonvulsants (i.e., phenytoin or carbamazepine), antihypertensives and drugs with hypotension as side effects (i.e., propranolol, terazosin, doxazosin, prazosin, atenolol, furosemide, nifedipine, verapamil, diltiazem, isosorbide dinitrate, lisinopril, amitriptyline, chlorpromazine, or procholorperazine), and psychotropic agents (i.e. , zolpidem, diazepam, alprazolam, or haloperidol).

The insurance enrollee category was used as a proxy measure of socio-economic status (SES). SES is an important prognostic factor for stroke and/or myocardial infarction [17], [18]. The patients were classified into two sub-groups: high individual SES (civil servants, regular or full-time paid personnel with a government affiliation, employees of privately owned institutions), and low individual SES (self-employed individuals, members of the farmers or fishermen's association, other employees, veterans, substitute service draftees, and members of low-income families) [19].

Urbanization level of residence is also associated with vascular events and were therefore included in our analysis [20], [21]. We recorded the level of urbanization as urban, and sub-urban (urbanization level 1–3), or rural (urbanization level 4–7).

### Statistical analysis

The SAS statistical package, version 9.2 (SAS Institute, Inc., Cary, NC), and SPSS version 15 (SPSS Inc., Chicago, IL) were used for data analysis. Pearson's chi-square test was used for categorical variables, demographic characteristics (age group and gender), comorbidities (hypertension, diabetes, coronary artery disease, hyperlipidemia and atrial fibrillation) and initially taking medications that may cause dizziness in ER patients with dizziness/vertigo and those without.

The 3-year vascular event-free survival rate was estimated using the Kaplan-Meier method. The cumulative risk of stroke or cardiovascular event was estimated as a function of time from initial treatment. Cox proportional hazard regression model with time-dependent covariate was used to calculate the risk of stroke or cardiovascular event for ER patients with dizziness or vertigo versus those without, after adjustments for age, gender, hypertension, diabetes, coronary heart disease, hyperlipidemia, atrial fibrillation, urbanization of residence, socioeconomic status, and initially taking medications. To find useful predictors, we constructed univariate and multivariable Cox models to explore the effect of age, gender, comorbidities, urbanization of residence, socioeconomic status and initially taking medications on the risk of vascular outcomes for patients with dizziness or vertigo. A *p<*0.05 was considered statistically significant in the regression models.

In order to test the validity of this study and specific association between dizziness/vertigo and vascular events, determining the risk of developing stroke or cardiovascular events in ER patients discharged home with a diagnosis of headache (ICD-9-CM 780.4) was further conducted. This dataset were selected from the Longitudinal Health Insurance Dataset for the period 2004–2006 as previously described. The outcomes measures were similar to those which was applied in dizziness and vertigo patients. Patients with any type of vascular events diagnosed before or during the index ER visit and those with specific diagnosis were excluded. 23165 patients (417 patients with headache and 22748 patients without a diagnosis of headache) who had visited ER and further discharged smoothly in 2004 were included. These patients were tracked for three years and further lined to the administrative data for the period 2004–2006 to estimate vascular event-free survival, with cases censored for patients who drew back guarantees from the National Health Insurance Program or were still robust without defined events at end of follow-up.

## Results

The distribution of demographic characteristics, selected morbidities and initially taking medications for the two cohorts is shown in Table 1. Dizziness/vertigo patients were significantly older and more likely to be female. They were also more likely to have hypertension, and diabetes, had lower socioeconomic status, and took anticonvulsants, antihypertensives and psychotropic agents than those without dizziness/vertigo.

**Table 1:** Baseline characteristics of the study population.

Variables	Entire population (n = 25757) n(%)	Dizziness or vertigo patients (n = 1118) n(%)	Patients without dizziness or vertigo (n = 24639) n(%)	*p* value
Gender				<0.001
Male	12451 (48.3)	395 (35.3)	12056 (48.9)	
Female	13306 (51.7)	723 (64.7)	12583 (51.1)	
Age group				<0.001
18–44	14279 (55.4)	373 (33.4)	13906 (56.4)	
45–54	4158 (16.1)	216 (19.3)	3942 (16.0)	
55–64	2632 (10.2)	171 (15.3)	2461 (10.0)	
65–74	2465 (9.6)	194 (17.4)	2271 (9.2)	
≧75	2223 (8.6)	164 (14.7)	2059 (8.4)	
Hypertension				<0.001
Yes	832 (3.2)	96 (8.6)	736 (3.0)	
No	24925 (96.8)	1022 (91.4)	23903 (97.0)	
Diabetes				<0.001
Yes	612 (2.4)	50 (4.5)	562 (2.3)	
No	25145 (97.6)	1068 (95.5)	24077 (97.7)	
Coronary heart disease				0.135
Yes	474 (1.8)	14 (1.3)	460 (1.9)	
No	25283 (98.2)	1104 (98.7)	24179 (98.1)	
Hyperlipidemia				0.450
Yes	18 (0.1)	0 (0.0)	18 (0.1)	
No	25739 (99.9)	1118 (100.0)	24621 (99.9)	
Atrial fibrillation				0.655
Yes	28 (0.1)	1 (0.1)	27 (0.1)	
No	25729 (99.9)	1117 (99.9)	24612 (99.9)	
Urbanization				0.063
Urban and suburban	20204 (78.4)	852 (76.2)	19352 (78.5)	
Rural	5553 (21.6)	266 (23.8)	5287 (21.5)	
Individual socioeconomic status				<0.001
High	12609 (49.0)	463 (41.4)	12146 (49.3)	
Low	13148 (51.0)	655 (58.6)	12493 (50.7)	
Anticonvulsants				0.001
Yes	350 (1.4)	31 (2.8)	319 (1.3)	
No	25407 (98.6)	1087 (97.2)	24320 (98.7)	
Antihypertensives				<0.001
Yes	4249 (16.5)	345 (30.9)	3904 (15.8)	
No	21508 (83.5)	773 (69.1)	20735 (84.2)	
Psychotropic agents				<0.001
Yes	3341 (13.0)	281 (25.1)	3060 (12.4)	
No	22416 (87.0)	837 (74.9)	21579 (87.6)	

**Figure 2:**
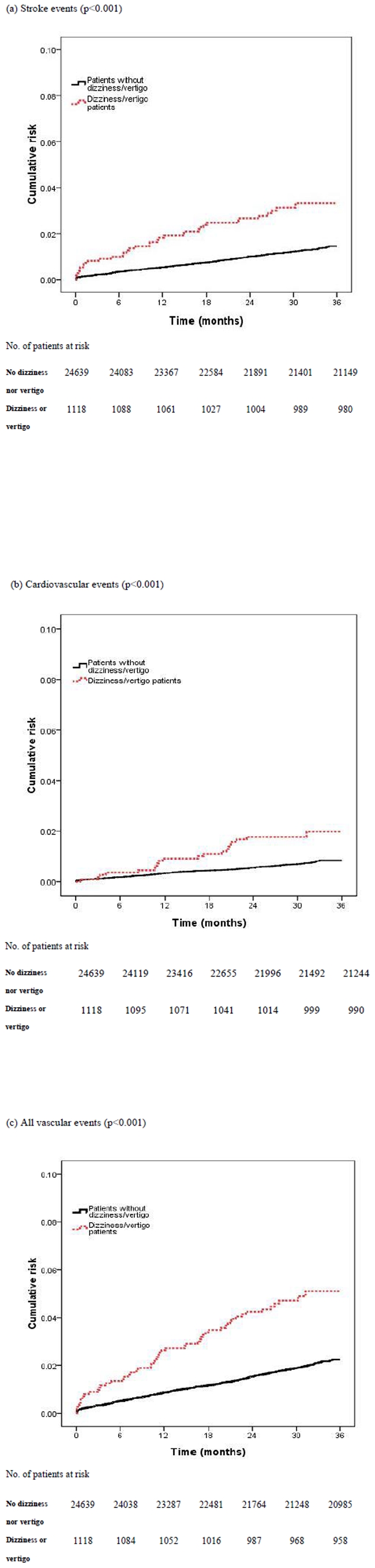
Cumulative risk of vascular events for the ER dizziness/vertigo patients and patients without dizziness/vertigo.

At the end of follow-up, 506 patients had vascular events, 52 (4.7%) in the patients with dizziness or vertigo and 454(1.8%) in those without. The six-month cumulative risk of vascular event, stroke, or cardiovascular events was 1.3% (95% Confidence interval [CI], 0.7–1.9), 1% (95% CI, 0.4–1.6), and 0.4% (95% CI, 0–0.8), respectively for the patients with dizziness or vertigo. In patients with dizziness or vertigo, the cumulative risk of vascular event, stroke, or cardiovascular events was 5.1% (95% CI, 3.7–6.5), 3.3% (95% CI, 2.0–4.5), and 2.0% (95% CI, 1.0–3.0), respectively at the end of follow-up (Table 2). Patients with dizziness or vertigo had an increased risk of vascular events (Figure 2). Table 3 shows in model with time interactions, the estimated of hazard ratios of dizziness/vertigo (HR = 0.65, 95% CI, 050–0.84; p = 0.001) were less than 1, which implied that the effects of dizziness/vertigo and comorbidities are much stronger at the beginning of the follow-up period and weaker after long-term follow-up. We further separated the follow-up time during the first year and during the 2^nd^ and 3^rd^ year. In Table 4, we see that the effect of dizziness or vertigo are much stronger for vascular events in the first year (HR = 2, 95% CI, 1.35–2.96). Among the ER patients with dizziness or vertigo discharged home, predictors of vascular events were analyzed using Cox regression model. In univariate analysis, age, diabetes, coronary artery disease, male gender, residence in rural area and initially taking antihypertensive drugs were associated with increased risk for vascular events. Increased age (HR = 1.06; 95% CI, 104–1.08), and coronary artery disease (HR = 5, 95% CI, 1.82–16) remained independent risk factors for vascular events in the multivariable model (Table 5).

**Table 2:** Cumulative risk of vascular events in patients with dizziness/vertigo and those without.

Variables	6 months		1-year		2-year		3-year	
	Events (%)	Risk (%) (95% CI)	Events (%)	Risk (%) (95% CI)	Events (%)	Risk (%) (95% CI)	Events (%)	Risk (%) (95% CI)
***Stroke***								
Patient without dizziness/vertigo	81 (0.3)	0.3 (0.3–0.3)	131 (0.5)	0.5 (0.5–0.5)	236 (1.0)	1.0 (0.8–1.2)	290 1.2)	1.4 (1.2–1.6)
Dizziness/vertigo patients	11(1.0)	1.0 (0.4–1.6)	20(1.8)	1.8 (1–2.6)	29 (2.6)	2.7 (1.7–3.7)	34 (3.0)	3.3 (2.0–4.5))
***Cardiovascular events***								
Patient without dizziness/vertigo	45 (0.2)	0.2 (0.2–0.2)	82 (0.3)	0.3 (0.3–0.3)	131 (0.5)	0.6 (0.6–0.6)	170 (0.7)	0.8 (0.6–1.0)
Dizziness/vertigo patients	4 (0.4)	0.4 (0–0.8)	10 (0.9)	0.9 (0.3–1.5)	19 (1.7)	1.8 (1.0–2.6)	20 (1.8)	2.0 (1.0–3.0)
***All vascular events***								
Patient without dizziness/vertigo	126 (0.5)	0.5 (0.5–0.5)	211 (0.9)	0.9 (0.7–1.1)	363 (1.5)	1.5 (1.3–1.7)	454 (1.8)	2.2 (2.0–2.4)
Dizziness/vertigo patients	15 (1.3)	1.3 (0.7–1.9)	29 (2.6)	2.6 (1.6–3.6)	46 (4.1)	4.2 (3.0–5.4)	52 (4.7)	5.1 (3.7–6.5)

**Table 3:** Hazard ratios of all vascular events among 25,757 patients.

Variables	Model without time interactions			Model with time interactions		
	Hazard ratio	95% CI	*p* value	Hazard ratio	95% CI	*p* value
Dizziness/Vertigo	1.62	1.21–2.17	0.001	4.60	2.40–8.33	<0.001
Age, per year	1.07	1.06–1.07	<0.001	1.31	1.29–1.33	<0.001
Hypertension	1.34	1.01–1.81	0.040	1.66	1.24–2.23	0.001
Diabetes	1.41	1.01–1.97	0.041	2.02	1.44–2.82	<0.001
Coronary heart disease	1.89	1.37–2.62	<0.001	2.33	1.66–3.26	<0.001
Atrial fibrillation	3.13	1.17–8.38	0.023	1.62	0.51–5.20	0.416
Male gender	1.27	1.07–1.52	0.008	1.11	0.92–1.33	0.284
Residence in rural area	1.18	0.98–1.44	0.089	1.07	0.88–1.32	0.492
Low individual SES	1.07	0.87–1.31	0.515	1.56	1.25–1.95	<0.001
Anticonvulsants	1.56	0.98–2.49	0.059	1.75	1.10–2.79	0.019
Antihypertensive drug	1.47	1.22–1.78	<0.001	1.44	1.17–1.76	<0.001
Psychotrophic drug	1.06	0.85–1.32	0.611	1.07	0.85–1.33	0.582
Dizziness/Vertigo × log(time)	-	-	-	0.65	0.50–0.84	0.001
Age × log(time)	-	-	-	0.93	0.92–0.93	<0.001

Abbreviation: 95% CI, 95% confidence interval.

**Table 4:** Hazard ratios of stroke, cardiovascular events, and all vascular events among the ER dizziness/vertigo patients and patients without dizziness/vertigo stratified by time; 240 vascular events in the first year of follow-up and 266 vascular events between 2^nd^ and 3^rd^ years.

Variables	Events in the 1^st^ year				Events between 2^nd^ and 3^rd^ years			
	No. of events	Hazard ratio*	95% CI	*p* value	No. of events	Hazard ratio*	95% CI	*p* value
***Stroke***								
Patient without dizziness/vertigo	131	1.00	–	–	157	1.00	-	-
Dizziness/vertigo patients	20	2.07	1.29–3.34	0.003	13	1.08	0.61–1.92	0.781
***Cardiovascular events***								
Patient without dizziness/vertigo	82	1.00	-	-	87	1.00	-	-
Dizziness/vertigo patients	10	1.94	1–3.76	0.051	10	1.72	0.89–3.34	0.107
***All vascular events***								
Patient without dizziness/vertigo	211	1.00	-	-	243	1.00	-	-
Dizziness/vertigo patients	29	2.00	1.35–2.96	0.001	23	1.31	0.85–2.02	0.216

Abbreviation: 95% CI, 95% confidence interval.

* Adjusted for age, gender, hypertension, diabetes, coronary heart disease, hyperlipidemia, atrial fibrillation, urbanization of residence, individual socioeconomic status and medication.

**Table 5:** Predictors of vascular events among ER patients discharged home with diagnosis of dizziness or vertigo (n = 1118).

Variable	Hazard ratio (95% CI)		
	All vascular events	Stroke	Cardiovascular events
**Univariate model** *			
Age, per year	1.07 (1.04–1.09)	1.07 (1.04–1.10)	1.06 (1.03–1.10)
Male gender	1.87 (1.08–3.23)	2.10 (1.04–4.12)	1.85 (0.77–4.45)
Hypertension	1.71 (0.77–3.80)	2.37 (0.98–5.71)	0.56 (0.08–4.20)
Diabetes	1.83 (0.66–5.07)	2.91 (1.02–8.25)	1.13 (0.15–8.47)
Coronary heart disease	7.61 (2.74–21)	5.18 (1.24–22)	9.34 (2.17–41)
Residence in rural area	2.02 (1.16–3.54)	1.76 (0.87–3.55)	2.62 (1.09–6.33)
Low individual SES	1.43 (0.80–2.54)	1.67 (0.80–3.48)	1.28 (0.51–3.20)
Anticonvulsants	2.25 (0.70–7.22)	2.29 (0.55–9.57)	1.90 (0.26–14)
Antihypertensive drug	2.35 (1.36–4.05)	1.43 (0.72–2.86)	4.34 (1.73–11)
Psychotrophic drug	1.64 (0.93–2.91)	0.94 (0.42–2.07)	3.83 (1.59–9.25)
**Multivariable regression model** *			
Age, per year	1.06 (1.04–1.08)	1.07 (1.04–1.10)	1.06 (1.03–1.10)
Male gender	1.95 (0.79–4.81)	1.58 (0.79–3.17)	1.95 (0.79–4.81)
Hypertension	0.87 (0.38–2.02)	1.33 (0.53–3.31)	0.35 (0.03–2.02)
Diabetes	1.78 (0.42–3.30)	1.78 (0.62–5.11)	0.79 (0.10–6.08)
Coronary heart disease	5.31 (1.82–16)	3.60 (0.80–36)	7.50 (1.63–34)
Residence in rural area	1.79 (0.98–3.28)	1.40 (0.65–3.03)	2.4 1(0.94–6.17)
Low individual SES	0.68 (0.36–1.27)	0.85 (0.39–1.89)	0.53 (0.19–1.47)
Anticonvulsants	2.05 (0.59–7.14)	2.04 (0.45–9.22)	2.45 (0.29–21)
Antihypertensive drug	1.52 (0.86–2.69)	0.99 (0.48–2.04)	2.29 (0.88–6.01)
Psychotrophic drug	1.38 (0.76–2.51)	0.81 (0.36–1.85)	3.3 (1.29–8.28)

Abbreviation: 95% CI, 95% confidence interval.

* History of hyperlipidemia and atrial fibrillation were not included in the regression model due to lack of data association between vascular outcomes and these two co-morbidities in dizziness/vertigo patients.

We further identify 417 ER patients discharged home with a diagnosis of headache and determine the risk of developing vascular events. They were more likely to be older, to be female, and had hypertension (Table 6). There was no statistically difference in risk of vascular events between patients with headache and patients without headache (Table 7).

**Table 6:** Baseline characteristics of ER patients with headache and patients without headache.

Variables	Entire population (n = 23165) n(%)	Headache patients (n = 417) n(%)	Patients without headache (n = 22748) n(%)	*p* value
Gender				<0.001
Male	11108(48)	132(31.7)	10976(48.3)	
Female	12057(52)	285(68.3)	11722(51.7)	
Age group				<0.001
<45	12721(54.9)	274(65.9)	12447(54.7)	
45–54	3758(16.2)	65(15.6)	3693(16.2)	
55–64	2408(10.4)	45(10.8)	2363(10.4)	
65–74	2264(9.8)	19(4.6)	2245(9.9)	
≧75	2014(8.7)	14(3.4)	2000(8.8)	
Hypertension				<0.001
Yes	797(3.4)	32(4)	765(3.4)	
No	22368(96.6)	385(96)	21983(96.6)	
Diabetes				0.057
Yes	547(2.4)	4(1)	543(2.4)	
No	22618(97.6)	413(99)	22205(97.6)	
Coronary heart disease				0.009
Yes	465(2)	1(0.2)	464(2)	
No	22700(98)	416(99.8)	22284(98)	
Hyperlipidemia				0.761
Yes	15(0.1)	0(0)	15(0.1)	
No	23150(99.9)	417(100)	22733(99.9)	
Atrial fibrillation				0.601
Yes	28(0.1)	0(0)	28(0.1)	
No	23137(99.9)	417(100)	22720(99.9)	
Urbanization				0.298
Urban and suburban	18201(78.6)	319(76.5)	17882(78.6)	
Rural	4964(21.4)	98(23.5)	4866(21.4)	
Individual socioeconomic status				0.583
High	11252(48.6)	197(47.2)	11055(48.6)	
Low	11913(51.4)	220(52.8)	11693(51.4)	

**Table 7:** Adjusted hazard ratios of vascular events among the ER headache patients and patients without headache (n = 23165).

Variables	No. of cases	(%)	Age-adjusted HR (95% CI)	Multivariate-adjusted* HR (95% CI)
***Stroke***				
Patients without headache	397	(1.7)	1	1
Headache patients	6	(1.4)	1.30 (0.58–2.91)	1.29 (0.57–2.90)
*p* value			0.527	0.537
***Cardiovascular events***				
Patients without headache	235	(1.0)	1	1
Headache patients	0	(0)	-	-
*P* value			-	-
***All vascular events***				
Patients without headache	614	(2.7)	1	1
Headache patients	6	(1.4)	0.79 (0.35–1.76)	0.83 (0.37–1.85)
*p* value			0.559	0.64

Abbreviation: HR, hazard ratio; 95% CI, 95% confidence interval.

* Adjusted for age, gender, co-morbidities, urbanization of residence, and individual socioeconomic status.

## Discussion

Our data showed that ER patients discharged home with a diagnosis of dizziness or vertigo incurred a two-fold higher risk for subsequent vascular events after adjusting for patient characteristics, comorbidities, and other confounding factors during the first year. Further studies are warranted for developing diagnostic and follow-up strategies.

The strengths of our study are its being a large population-based study (n = 25,757), nearly complete follow-up of any hospitalization for vascular events among the whole study population (99%) and routine monitoring of accuracy of diagnosis by the National Health Insurance Bureau of Taiwan. We see the association between ER visit for headache and vascular outcomes in order to test the specific association of dizziness/vertigo and vascular events. There was no statistically association between headache and vascular events. The specific association of dizziness/vertigo and vascular events appeared to be specific to dizziness/vertigo but not headache. One previous study showed 180-day cumulative incidence of vascular event, cerebrovascular event, or cardiovascular event was 0.93%, 0.63%, and 0.32%, respectively and revealed that most of the vascular events developed within the first month [8]. Our series revealed the six-month cumulative risk of vascular event, stroke, or cardiovascular events was 1.3%, 1%, and 0.4%, respectively. The risk for vascular events remained significantly higher during the first year follow-up and became insignificant between second and third years. In another follow-up study, up to 5% of major vascular events developed within six months. However, up to 18% loss to follow-up may prevent us to interpret these results correctly [7]. Our results suggest that ER patients discharged home with a diagnosis of dizziness or vertigo have a higher risk for stroke, cardiovascular events and all vascular events, compared to those without dizziness/vertigo after adjusting patient characteristics, co-morbidities, and other confounding factors.

Our finding of up to 4.7% of vascular events among patients with dizziness/vertigo over 3-year follow-up may justify utilizing more intensive evaluation to identify and treat serious cases. We found a similar pattern of cumulative risks for vascular events as previously reported, with more early cerebrovascular events, but not more early cardiovascular events [8]. Early vascular events within several months after discharge from the ER may be attributable to conditions reported at the ER visit. The later flattening in the curve may reflect the background rate of cardiovascular risk [8]. Dizziness is a vague term which may refer to vertigo, near-fainting, psychological problems, ocular-vestibular disease or disequilibrium [22]. One previous study found that most dizziness in ER patients was attributable to medical causes [23]. When considered in aggregate, general medical diagnoses were more common than otovestibular ones, and nearly half of the medical disorders diagnosed were cardiovascular. This observation indicates that patients with dizziness or vertigo may have underlying cardiovascular disease. On the other hand, dizziness or isolated vertigo which results from transient ischemia to the vestibular labyrinthine is a common symptom in nonfocal transient neurological attacks. Our results are in agreement with those of Bos et al. and may be partially explained by their observations [4]. They challenge the strong but unfounded conviction that nonfocal transient neurological attacks are harmless and first reported their association with increased risk for dementia (HR, 1.59; 95% CI, 1.11–2.26) and stroke (HR, 1.56; 95% CI, 1.08–2.28). Similarly, in our series, ER patients discharged home with a diagnosis of dizziness or vertigo also had increased risk for vascular events (HR, 2; 95% CI, 1.35–2.96) during the first year follow-up.

Widespread uncertainty involved in decision related to dizziness/vertigo presentations still exists in the ER even after lengthy evaluations and extensive use of diagnostic tests, including neuroimaging [23]. We found that increasing age and coronary artery disease were independent risk factors for stroke and all vascular events among ER patients discharged with a diagnosis of dizziness or vertigo. For these increased risk patients, emergency neurological/cardiac consultation or referral to out-patient clinics might be considered because of the possibility of misdiagnosis of dangerous disorders in the ER. Furthermore, increased risk patients need to be educated to identify the warning signs of stroke, transient ischemic attack and myocardial ischemia. Given that older people are most vulnerable to vascular events, it is particularly important to maximize the potential for early recognition and early medical intervention in this group [24], [25].

After symptomatic treatment in the ER for the dizziness or vertigo, patients need watchful attention to prevent vascular events for several years. Although the incidence is relatively low, cardiovascular and cerebrovascular events remain the main causes of death and adult disability worldwide. If misdiagnosed, some of these patients can have poor outcomes. Widely used diagnostic strategies with categories such as vertigo, lightheadedness, and disequilibrium are difficult to apply in the ER setting for acute decision making [23]. Given this diagnostic uncertainty, identification of increased risk patients is important. No guidelines currently exist for the primary prevention of stroke or cardiovascular events in increased risk vertigo or dizziness patients, and studies evaluating the role of antiplatelet agents for such patients are warranted.

This study has several limitations. First, the diagnosis of dizziness or vertigo, stroke, myocardial infarction, ventricular arrhythmia and any other co-morbid conditions are completely dependent on ICD-9-CM codes. Due to using deidentified administrative data, a direct validation of accuracy of diagnosis and outcomes were not possible with chart review. Nonetheless, the National Health Insurance Bureau of Taiwan randomly reviews the charts and interviews patients to verify the accuracy of diagnosis. Hospitals with outlier chargers or practice may undergo an audit, with subsequent heavy penalties for malpractice or discrepancies. We used previously validated algorithms with high accuracy to identify outcomes from discharge diagnosis codes. The accuracy of the National Health Insurance Research Database in recording ischemic stroke diagnoses is as high as 95% [26]. Second, detailed information on myocardial infarction, unstable angina, ventricular arrhythmia, or strokes cannot be exactly extracted from ICD-9-CM codes, which prevents further sub-group analysis. Further studies linking administrative data and primary hospitalization information are warranted. Third, for the analysis of the initially taking medications that may cause dizziness or vertigo, binary event of “yes” versus “no” was recorded. The length of time and dosage which patients were on these medications were not clearly defined. More detailed research for analyzing the impact of these medications is necessary in the future. Fourth, only dizziness or vertigo patients who were considered safe enough to be discharged home were included in our series. Our results were unable to be generalized to patients with dizziness/vertigo and specific diagnosis or those who were further admitted to ward later.

In summary, this study reveals that ER patients with dizziness or vertigo have a increased risk of subsequent vascular events and that their likelihood of developing stroke or cardiovascular events during the first year is 2 times higher than that of those without dizziness or vertigo. Although the incidence is relatively low (95.3% of patients remain event-free at 3 years), efforts to prevent vascular events after the onset of dizziness or vertigo is worthy of further study.
